# Baseline thrombocytopenia complicated by recurrent episodes of transient severe thrombocytopenia following infections in an adult woman with a non involuting congenital hemangioma – a case report

**DOI:** 10.1186/2052-1839-13-7

**Published:** 2013-06-13

**Authors:** Mitrakrishnan C Shivanthan, Bandula Wijesiriwardena, Indira S Wijesiriwardena

**Affiliations:** 1 National Hospital of Sri Lanka, Regent Street, Colombo 7, Sri Lanka; 2 Lanka Hospitals, Colombo 5, Sri Lanka; 3 Faculty of Medicine, University of Sri Jayawardenapura, Nugegoda, Sri Lanka

**Keywords:** Hemangioma, Thrombocytopenia, Severe thrombocytopenia, Infection, Platelet

## Abstract

**Background:**

Congenital hemangiomas are benign abnormal proliferation of blood vessels. Noninvoluting congenital hemangiomas are a rare variant which persist, and may become bigger. Hemangiomas are known to be associated with thrombocytopenia, microangiopathic hemolytic anemia and Kasabach-Merritt phenomenon. Kasabach-Merritt phenomenon is characterized by consumptive coagulopathy with microangiopathic haemolyic anemia and thrombocytopenia. Platelet sequestration in the hemangioma or increased destruction which may either be immune or non immune are also further contributors to thrombocytopenia.

**Case presentation:**

A 45 year old female with a non involuting hemangioma and baseline thrombocytopenia was observed to develop repeated episodes of transient severe thrombocytopenia associated with a variety of infectious conditions. Laboratory investigations suggested a peripheral mechanism. Platelet counts always returned to baseline levels on resolution of the precipitating infection.

**Conclusion:**

The authors report this phenomenon as the first reported case of baseline thrombocytopenia complicated by recurrent episodes of transient severe thrombocytopenia following infections associated with a non involuting congenital hemangioma. The observations made in this patient were unique and hitherto unreported in medical literature. Both peripheral sequestration and destructive consumption were considered likely. Consumptive mechanisms were likely to encompass either or both immune and non immune causes. Further studies are needed to establish the precise pathogenesis.

## Background

Congenital hemangiomas are benign abnormal proliferation of blood vessels [1]. Noninvoluting congenital hemangiomas are a rare variant of cutaneous vascular tumors of intrauterine onset which persist - either unchanged or with slight enlargement [2]. Hemangioma-thrombocytopenia syndrome associated with microangiopathic hemolytic anemia has been documented in children. The Kasabach-Merritt phenomenon was described first in 1940 as a syndrome of consumptive coagulopathy, in patients with capillary hemangiomas characterized by microangiopathic haemolyic anemia and thrombocytopenia [3]. Rapidly involuting congenital haemangiomas are known to be associated with fluctuating thrombocytopenia and coagulopathy [4]. Thrombocytopenia is a complication seen in adult patients with large or multiple hemangiomas [5]. This phenomenon may occur due to sequestration in the hemangioma or increased destruction which may be due to either immune or non immune causes [6]. Chronic immune thrombocytopenia is known to occur in infections [7], and acute immune mediated thrombocytopenia has been demonstrated in certain infections and hypersensitivity states [8-11]. Thrombocytopenia associated with infections has been known to parallel the severity of illness [12]. However literature search yielded no similar case with baseline thrombocytopenia complicated by recurrent episodes of transient severe thrombocytopenia following infections in a patient with a non involuting congenital hemangioma.

## Case presentation

A 45 year old single woman with a non regressing congenital vascular malformation of the right arm and upper chest, presented with a 3 day history of high fever, chills, dysuria, hematuria and right loin discomfort. Her urine output was normal and she had no spontaneous bleeding manifestations or features of uremia. A marked drop in her platelet counts compared to recent baseline levels had been detected by her primary care physician following the onset of symptoms.

Her past medical history was significant for persistent low baseline platelet counts in the region of 100,000/mm3 with episodic reduction to values as low as 30,000/mm3 following infections. She had been extensively and repeatedly investigated from the time the low baselines platelet counts were detected at the age of 28, however no definite cause was identified. On direct questioning she denied spontaneous mucocutaneous bleeding in the past. However her past medical history was significant for an abandoned plastic surgical procedure after developing a haematoma surrounding the preoperative intravenous cannulation site with a corresponding platelet count of 47,000/mm3. She had also been managed conservatively for uterine adenomyosis with depot provera from the age of 45 due to concerns of high risk of perioperative bleeding.

Detailed evaluation was negative for symptoms attributable to anemia, connective tissue disease and liver disease. History of medications was negative for agents associated with thrombocytopenia.

**Figure 1:**
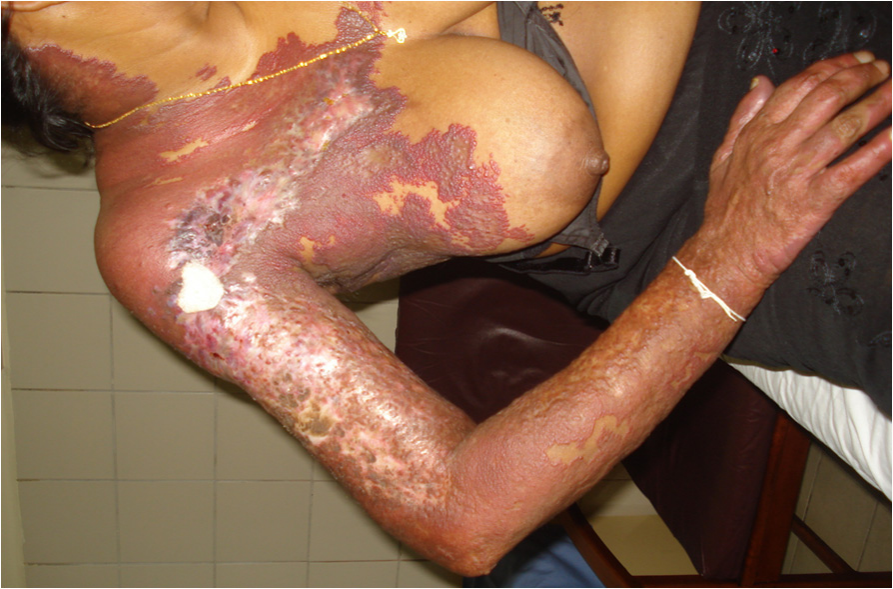
Non involuting hemangioma with ulcerated areas.

Examination revealed an ill looking lady with a temperature of 100.4 degrees Fahrenheit. She had a giant hemagioma involving the entire right arm, right lateral aspect of the neck and both the anterior and posterior aspect of the chest extending up to the 4th intercostal space (Figure 1). The hemangioma had small areas which were ulcerated and inflamed as well as healed scars. A soft bruit was appreciated on auscultation over the hemangioma. She had no petichiae or ecchymoses and her conjunctivae were pink. No lymphadenopathy was detected.

Cardiovascular examination was remarkable only for a pulse rate of 104/min. The blood pressure was 100/60 mm Hg. Abdominal examination revealed mild right loin tenderness without any organomegaly. Clinical pelvic examination was not performed respecting the patient’s wishes. Respiratory system and nervous system examination were both unremarkable.

The urine full report demonstrated 15–20 pus cells and 30–40 red cells per high powered field. Complete blood count on admission showed a hemoglobin of 11 g/dl with a neutrophil leukocytosis (WBC count of 17,000/mm3 – neutrophils 88%, lymphocytes 10%). The platelet count was 27,000/mm3. C reactive protein was elevated at 196 mg/dl (reference upper limit - 6 mg/dl). Serial complete blood counts done on days 4 & 5 of the illness revealed a progressive drop in platelet counts to a nadir of 15,000/mm3. Blood film showed severe thrombocytopenia with toxic neutrophils. Schistocytes suggestive of microangiopathic haemolytic anemia were notably absent. Her serum creatinine was mildly elevated at 1.52 mg/dl (reference upper limit - 1.3 mg/dl). Other biochemical parameters including uric acid, electrolytes and liver profile were all within normal limits. Both blood culture and urine culture yielded gram negative Escherichia coli with identical overlapping antibiotic sensitivity patterns. The wound swab from the ulcerated areas of the hemangioma became positive for Staphylococcus aureus sensitive to both penicillin and cloxacillin. Dengue NS1 antigen was negative. Ultrasonic examination of the abdomen excluded splenomegaly, features of portal hypertension and chronic liver disease. HIV, ANA, antiphospholipid antibodies, antiplatelet antibodies and Coombs test were all negative.

Background consumptive coagulopathy was excluded with normal baseline INR, APTT, D-dimers by modified ELISA and fibrinogen levels. There was also no laboratory evidence of acute derangement of the coagulation profile in relation to the worsening of thrombocytopenias triggered by infections.

In depth perusal of her previous admission records confirmed repeated episodes of severe thrombocytopenia (<30,000/mm3) precipitated with a variety of infections with return to baseline values of approximately 100,000/mm3 on resolution of the illness (Table 1). Despite extensive investigations no cause or basis had ever been identified in her to explain the phenomenon. The last episode of severe thrombocytopenia was triggered by an upper respiratory tract infection which had occurred 4 months preceding her current illness. Community acquired infections and wound sepsis complicating ulcerated areas over the hemangioma were the commonest precipitants identified.

**Table 1:** Platelet count timeline in relation to clinical condition

**Date**	**Platelet count × 10**^**3/**^**uL**	**Cliinical status**
13 February 2002	85	Baseline
20 May 2003	75	Baseline
01 September 2003	50	Respiratory tract infection
08 September 2003	47	Surgery abandoned – Cannula site bleed
10 October 2003	22	Fever- Source / Cause? Viral
10 February 2004	33	Gasteroenteritis
20 February 2004	88	Convalescent
03 March 2006	123	Baseline
01 July 2006	23	Tonsillitis
11 July 2006	86	Baseline
14 July 2006	78	Baseline
07 August 2006	171	Baseline
25 November 2006	38	Viral fever
01 December 2006	88	Convalescent
26 December 2006	86	Baseline
23 January 2007	134	Baseline
01 December 2007	24	Otitis media
22 December 2007	88	Baseline
10 April 2009	22	Skin sepsis
14 April 2009	71	Convalescent
27 April 2009	143	Baseline
08 August 2010	52	Skin sepsis
12 August 2010	72	Baseline
19 August 2010	90	Baseline
02 May 2011	130	Baseline
02 July 2012	48	Upper respiratory tract infection
03 July 2012	46	Upper respiratory tract infection
04 July 2012	60	Upper respiratory tract infection
10 August 2012	92	Baseline
01 October 2012	61	Urinary & skin sepsis
02 October 2012	27	Urinary & skin sepsis
03 October 2012	15	Urinary & skin sepsis
06 October 2012	68	Urinary & skin sepsis
07 October 2012	78	Convalescent
14 October 2012	101	Baseline

Bone marrow studies done during a recent episode of severe thrombocytopenia had demonstrated a normal marrow including megakaryopoiesis, whilst the peripheral blood smear had shown evidence of thrombocytopenia and toxic left shift in the neutrophils which were considered to be consistent with a peripheral cause for thrombocytopenia on a background of ongoing infection. Repeated therapeutic trials with steroids had shown no improvement in the thrombocytopenia during episodes of infection and a rise in platelet counts was always temporally related to the resolution of the infection rather than steroid therapy. The baseline thrombocytopenias were not severe enough to warrant continued steroid therapy. However the episodic thrombocytopenias with infections had initially been treated with steroids. Platlet count trends during infections showed that the thrombocytopenia seemed to improve with the resolution of the causative infection rather than steroids. This was observed on many occasions in the patient and after the initial few trials no further steroid therapy was attempted. Thrombocytopenia was seen even on several occasions where the patient presented with untreated infections where she had neither been prescribed or used any medications and hence a drug induced cause was deemed unlikely. Acetaminophen was used by the patient unrelated to infections as an analgesic especially in relation to painful menstruation and no alteration in baseline platelet counts attributable to acetaminophen were seen.

Her current illnesswas treated with a combination of intravenous levofloxacin and clindamycin which resulted in complete defervescence by the 3rd day of hospitalisation. When she was discharged on the 3rd day post admission, her platelet counts had risen to 79,000/mm3 with improvement in serum creatinine to 1.2 mg/dl and the urine full report was free of any evidence of persistent urinary sepsis. She was discharged on oral co amoxiclav and clindamycin for a further period of one week and advised to continue oral penicillin indefinitely to prevent wound sepsis.

## Discussion

Congenital hemangiomas are benign abnormal proliferation of blood vessels [1]. Hemangiomas arise from mesenchymal tissue composed of multiple vascular channels lined with a single layer of endothelium on a scaffolding of fibrous connective tissuewhich frequently proliferate early in life before regressing in size. Vascular malformations despite many similarities in architecture to hemangiomas, differ by the presence of dysplastic endothelium and lack of regression. Congenital hemangiomas despite being present at birth often become clinically evident only later. Vascular malformations are named after the vascular element they most closely resemble namely capillary, venous, and lymphatic [13]. Rapidly involuting (RICH) and non-involuting (NICH) “congenital” hemangiomas appear to be histopathological hybrids showing features of both vascular tumor and malformation, and both entities are believed to lie within the same spectrum [14].

Noninvoluting congenital hemangiomas are rare variants of cutaneous vascular tumors of intrauterine onset without sex prevalence and they are described as well-circumscribed, plaque-like or slightly bossed lesions which never disappear and persist either unchanged or with slight expansion [2].

In a case series by Jin et al. magnetic resonance imaging, computed tomographic angiography and digital subtraction angiography of non involuting congenital hemangioma gave findings similar to radiological findings of common infantile hemangiomas [15].

The platelet count in adults ranges from 150,000 to 450,000/mm3, with mean values of 237,000 and 266,000/mm3 in males and females, respectively [16]. Thrombocytopenia is defined as a platelet count of less than 150,000/mm3. Thrombocytopenia may be caused by increased peripheral destruction, sequestration or reduced production. A detailed description of the causes thrombocytopenia is not within the scope of this case report, as a multitude of causes including many states of infection could be possible aetiologies [6].

Clinical observation of repeated episodes of severe thrombocytopenia during a succession of varieties of infections temporally establishes the relationship between the phenomenon of severe thrombocytopenia and infections in this patient The authors excluded common and likely alternative causes of thrombocytopenia in the subject including dengue fever, drugs, portal hypertension and marrow pathologies and therefore the phenomenon is explained by either sequestration or increased destruction within the hemangioma which may either be immune or non immune [6]. This is further supported by the combined findings of the blood film and bone marrow trephine biopsy done during a similar episode in the past which showed thrombocytopenia and normal megakaryopoiesis respectively. The chronic thrombocytopenia is likely due to due to chronic consumption within the hemangioma. A baseline state of inflammation within the hemangioma with aggravation due to infections causing increased consumption could be postulated however demonstrating or disproving this requires molecular and microscopic studies which are not feasible for ethical reasons. Sequestration causing thrombocytopenia is also unlikely as an explanation for the acute thrombocytopenia with infection though such an explanation cannot be discounted as the reason for the baseline state of low platelets. Radiolabelled platelet tracer studies are not possible in the clinical setting where the authors encountered the patient.

In patients without hemangiomas chronic immune thrombocytopenia is known to occur in infections [7], and acute immune mediated thrombocytopenia has been demonstrated in certain infections and hypersensitivity states [8-11]. Thrombocytopenia associated with infections has been known to parallel the severity of illness [12]. It was postulated that this patient who had a baseline thrombocytopenia due to sequestration and destruction within the hemangioma most likely developed superimposed immune mediated destruction of platelets triggered by a variety of infective conditions.

Hemangioma-thrombocytopenia syndrome associated with microangiopathic hemolytic anemia has been documented in children. The Kasabach-Merritt phenomenon was first described in 1940 as a syndrome of consumptive coagulopathy, with capillary hemangiomas having clearly demonstrable microangiopathic haemolyic anemia and thrombocytopenia [3]. Rapidly involuting congenital haemangioma is known to be associated with transient thrombocytopenia and coagulopathy [4]. Although thrombocytopenia has been documented in adult patients with large or multiple hemangiomas s no reports were found in literature documenting repeated episodes of severe thrombocytopenia triggered by a variety of successive infectious conditions in a patient having a non involuting congenital hemangioma with baseline thrombocytopenia.

## Conclusion

The authors report this case as a novel observation and suggest that further studies are needed to establish the phenomenon and pathogenesis in cohorts with non involuting congenital hemangiomas.

## Consent

Written informed consent was obtained from the patient for publication of this case report and any accompanying images. A copy of the written consent is available for review by the editor of this journal.

## Competing interests

The author(s) declare that they have no competing interests.

## Authors’ contributions

All authors were involved in the clinical management of the patient, literature survey and drafting of the manuscript. All authors read and approved the final manuscript.

## Pre-publication history

The pre-publication history for this paper can be accessed here:

http://www.biomedcentral.com/2052-1839/13/7/prepub
